# Molecular Determination of *Fasciola Spp*. Isolates from Domestic Ruminants Fecal Samples in the Northwest of Iran

**Published:** 2017

**Authors:** Abbas IMANI BARAN, Habib CHERAGHI SARAY, Farzad KATIRAEE

**Affiliations:** Dept. of Pathobiology, Faculty of Veterinary Medicine, University of Tabriz, Tabriz, Iran

**Keywords:** *Fasciola*, Egg, Ruminant, PCR-RFLP, Iran

## Abstract

**Background:**

*Fasciola* species are the main causes for fascioliasis with great financial losses and are among the most important food/water-borne parasites worldwide. The basic proceedings such as epidemiology and effective control of fascioliasis rely mainly on precise identification of *Fasciola* species. The present study was conducted to determine the *Fasciola* species in ruminant fecal samples from East Azerbaijan Province in Iran.

**Methods:**

Overall, 2012 fecal samples were collected and processed initially for microscopic examination of *Fasciola* eggs in 2014–15. Then, recovered eggs were subjected to molecular identification. A fragment of 618 bp of the 28S rRNA gene pertaining to *Fasciola* genus was amplified under PCR. The amplified fragment was restricted by fast digest *Ava* II enzyme in order to a Restriction Fragment Length Polymorphism.

**Results:**

Based on microscopic examination, 72 samples were infected, from which, 10 and 62 cases pertained to cattle and sheep samples respectively. Based on RFLP, the PCR products restricted by the *Ava* II restriction enzyme produced 529 bp fragments only. According to the positive controls, all restriction patterns were related to *Fasciola hepatica*, while no restriction patterns were linked to *F. gigantica*.

**Conclusion:**

Based on PCR-RFLP, *F. hepatica* was dominant species in animals of the studied areas and no evidence of *F. gigantica* was observed. Therefore, further field studies to verify these results are suggested.

## Introduction

*Fasciola* species, *F. hepatica,* and *F. gigantica* are the main causes for fascioliasis in various animals, specially ruminants, considered as one of the most important parasitic diseases with substantial economic losses owing to fertility disorders, reduction of growth and animal production, condemnation of a large number of infected livers in abattoir inspections, high morbidity and mortality, as well as great cost of veterinary services for diagnosis or treatment (1, 2). The financial losses caused by fascioliasis in Iranian animal farming are approximately thousands of dollars, annually (3). Moreover, fascioliasis, as an important emerging food and water-borne parasitic zoonosis, take places in human communities and it is considered as the most significant public health problem in several countries (1, 2) such as Iran (4). In the worldwide, *F. hepatica* mainly distributes in temperate climates and *F. gigantica* is commonly found in tropical areas, although these two species have an overlapping distribution on subtropical zones (1, 2). Additionally, the intermediate forms of *Fasciola* have been demonstrated based on morphometric and molecular documented indices in some areas of the world, such as Iran (5), Korea (6), Japan (7) and Egypt (8). These intermediate forms are difficult to identify accurately (9). Recently, due to various factors such as climatic variations and environmental modifications made by humans, the incidences of fascioliasis are increasing in some areas of the world (10, 11).

The basic proceedings such as epidemiology and effective control of fascioliasis rely mainly on precise identification of *Fasciola* species (12). Based on traditional approaches, detection of fascioliasis in living ruminants depends on the microscopic observation of *Fasciola* eggs in the fecal samples of infected animals (1). However, these conventional methods of identifying eggs cannot guarantee the accurate identification and intra-specific differences of *Fasciola* species, as eggs have a similar morphology to each other and are even morphologically indistinguishable from those of the other trematode (2, 13). Nowadays, molecular approaches as a powerful diagnostic method are widely used to study various parasitic infections such as fascioliasis and recently, Iranian parasitologists have considered using this reliable tool for detection of diversity and abundance of *Fasciola* spp infections in livers of slaughtered final hosts (4, 5, 12, 14–18) and in lymnaeid snails as intermediate hosts (3).

Several researchers have demonstrated infection by parasites such as *Echinococcus* spp (19), *Toxocara* spp (20), *F. hepatica* (1, 21), *Ostertagia ostertagi* (22), *Oesophagostomum bifurcum* and *Necator americanus* (23), and *Opisthorchis viverrini* (24) using PCR assay of feces. There is no evidence regarding molecular discrimination of *Fasciola* species agents in fecal specimens from living ruminants in Iran, though there are many studies that deal with the genetic characterization of adult forms of *Fasciola* species from slaughtered animals in different regions of Iran (4, 14–16).

This study was initiated to determine the discrimination of *Fasciola* species in fecal samples from cattle and sheep in the northwest of Iran by using PCR-RFLP analysis of 28S rRNA gene.

## Materials and Methods

### Sampling and Microscopic Examination

**Fig. 1:**
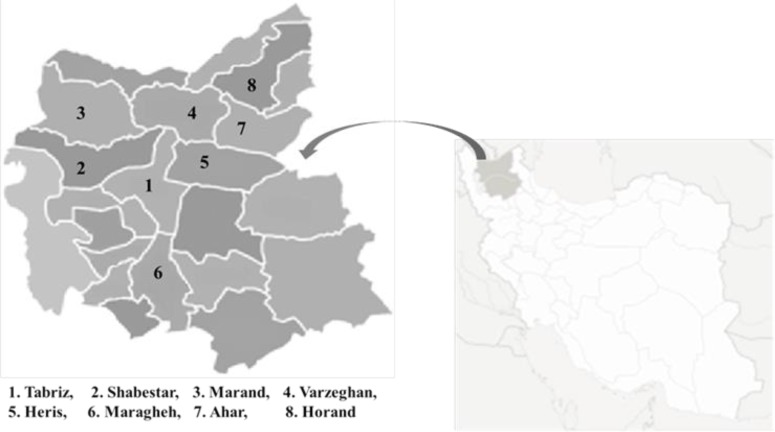
Location of sampling sites characterized by numbers on East Azerbaijan Province map, northwestern Iran

In this field study, from Nov 2014 to May 2015, a total of 2012 fresh fecal samples were collected from the rectum of ruminants (including 230 cattle and 1782 sheep) from various geographical regions of East Azerbaijan Province in Iran (Fig. 1, Table 1).

**Table 1:** The results of microscopic and molecular examinations on ruminant’s fecal samples from East Azerbaijan province

**Animal**	**No. of Fecal samples**	**No. of microscopically positive (%)**	**PCR-RFLP diagnosis**
Cattle	230	10 (4.35)	*F. hepatica*
Sheep	1782	62 (3.48)	*F. hepatica*
Total	2012	72 (3.58)	*F. hepatica*

Each sample was individually placed into plastic containers and finally, transported under low-temperature condition (<10 °C) to the Parasitology Laboratory of Faculty of Veterinary Medicine, University of Tabriz. At the end of the sampling period all fecal samples used for microscopic examination of *Fasciola* eggs were processed by means of standard floatation method (25).

### DNA Extraction

**Fig. 2:**
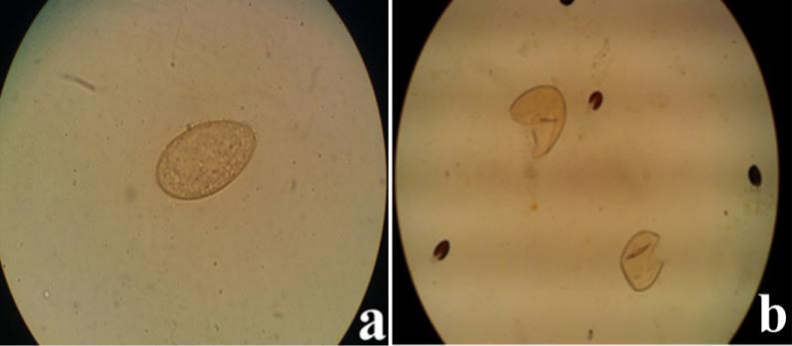
**a**) *Fasciola* egg before disruption; **b**) Disrupted *Fasciola* egg after freezing and thawing

The *Fasciola* eggs were recovered from infected fecal samples by floating 3 gr of each fecal sample in any time with slight modification. The recovered eggs were then re-suspended in PBS to give a pool of eggs. Then, by adding proteinase K, SDS 1%, and glass beads, the resultant mixtures were frozen and thawed several times in order to disturb the egg shells (Fig. 2).

Afterward, the mixtures were centrifuged at 12000 rpm for 15 min, supernatant was discarded and 300 μl of sediment was used for the extraction of total genomic DNA using Phenol-Chloroform method (1, 26). The extracted DNA was stored at −20 °C until future use.

### Polymerase Chain Reaction (PCR)

A fragment of 618 bp of the 28S rRNA gene pertaining to *Fasciola* species was amplified under PCR reaction by using a pair specific primer (Sense: 5′-ACG TGA TTA CCC GCT GAA CT-3′ and Antisense: 5′-CTG AGA AAG TGC ACT GAC AAG-3′) (27). The PCR was performed according to our previous study (3). Finally, 10 microliter from each PCR product as well as positive controls (PCR mix containing the DNA of adult form of *F. hepatica* collected from infected livers in Tabriz industrial abattoir, and the DNA sample extracted from adult form of *F. gigantica* provided from the archive of parasite specimens preserved in the Parasitology Laboratory of Faculty of Veterinary Medicine, Urmia University) and negative controls (PCR mix without *Fasciola spp*. DNA) were analyzed through electrophoresis on 1.5% agarose gel for approximately 1.5 h at 90 V, and imagined by staining with 1% ethidium bromide (3).

### RFLP analysis

To differentiate *Fasciola* species, an RFLP analysis was carried out on PCR products of *Fasciola* egg DNA. The restriction enzyme *Ava* II was selected to digest the DNA of *F. hepatica* and *F. gigantica* producing the three expected restriction fragment sizes of 529, 62 and 27 bps for *F. hepatica* and three fragment sizes of 322, 269, and 27 bps for *F. gigantica* (27). According to our previous study with slight modification, those required components and volumes for the restriction digestion were applied and due to using fast digest *Ava* II, the reaction tubes were placed in an incubator at 37 °C for 1hour. Ultimately, the restricted PCR products were electrophoresed on 2% agarose gel and imagined by ethidium bromide staining (3).

## Results

**Fig. 3:**
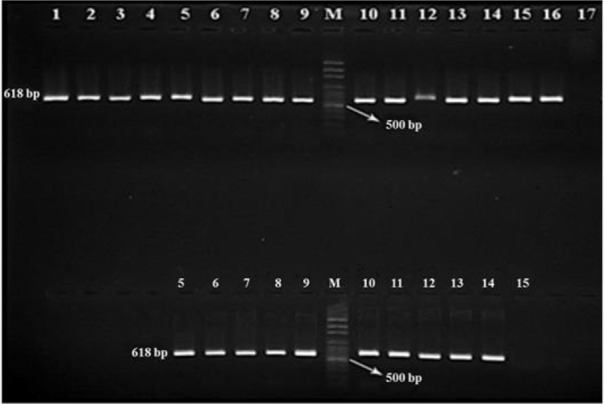
PCR products electrophoresis of 618 bp fragment of 28S rRNA gene pertaining to *Fasciola* species eggs Top row: wells 1–9 and 12–16 (Samples from sheep); bottom row: wells 5–9 and 12–14 (Samples from cattle); M: 100 bp DNA ladder; well 10: *F. hepatica* (Positive control); well 11: *F. gigantica* (Positive control); wells 17 (above) and 15 (below): Negative control

**Fig. 4:**
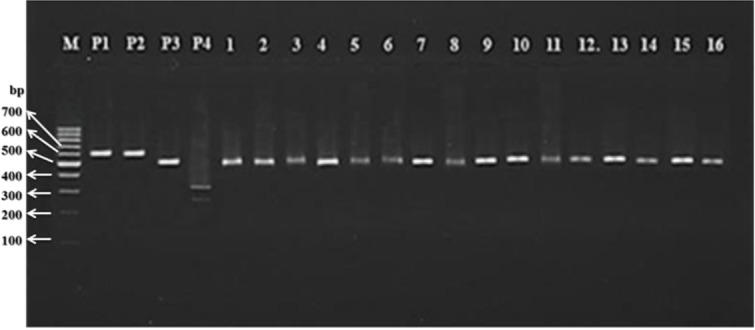
RFLP pattern of 618 bp fragment of 28S rRNA gene pertaining to *Fasciola* spp. eggs from sheep after digestion with *Ava* II: wells 1–16 (529 bp bands pertaining to *F. hepatica*); well P1 (618 bp band of *F. hepatica*, positive control from PCR product); well P2 (618 bp band of *F. gigantica*, positive control from PCR product); well P3 (529 bp band of *F. hepatica*, positive control after digestion with *Ava* II); well P4 (322 and 269 bp bands of *F. gigantica*, positive control after digestion with *Ava* II); M: 100 bp DNA ladder

**Fig. 5:**
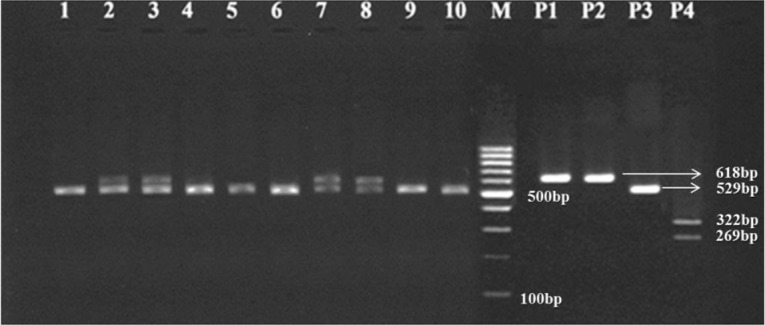
RFLP pattern of 618 bp fragment of 28S rRNA gene pertaining to *Fasciola* eggs from cattle after digestion with *Ava* II: wells 1–10 (529 bp bands pertaining to *F. hepatica*); well P1 (618 bp band of *F. hepatica*, positive control from PCR product); well P2 (618 bp band of *F. gigantica*, positive control from PCR product); well P3 (529 bp band of *F. hepatica*, positive control after digestion with *Ava* II); well P4 (322 and 269 bp bands of *F. gigantica*, positive control after digestion with *Ava* II); M: 100 bp DNA ladder

Based on microscopic examination of fecal samples and considering the morphological characterization of *Fasciola* egg, 72 (3.58%) out of 2012, samples were positive microscopically, from which, 10 and 62 cases pertained to cattle and sheep samples respectively (Table 1). For molecular diagnosis, all *Fasciola* eggs recovered from infected fecal samples were individually subjected to DNA extraction and PCR from which a 618 bp fragment of the 28S rRNA gene with specific primers pertaining to *Fasciola* genus was amplified (Fig. 3). Based on RFLP, the PCR products restricted by the *Ava* II restriction enzyme produced 529 bp fragments only (Fig. 4, 5). According to the positive controls, all restriction patterns were related to *F. hepatica*, while no restriction patterns were linked to *F. gigantica* (Table 1).

## Discussion

Fascioliasis is taken into account as an emerging and re-emerging food-born zoonosis in many regions of the world (1, 21, 28). In Iran, two important incidences of fascioliasis occurred in the 1990s in Guilan Province located in the north of Iran and are considered unprecedented, as they were the most severe outbreaks of fascioliasis on record, resulting from those more than 10000 people were affected. In each incidence, the features of this disease were altered and its significance became evident (29). Therefore, it seems necessary to be used molecular diagnostic methods for studying epidemiological aspects of animal and human fascioliasis in a developing country like Iran.

In the past, identification of endoparasites through demonstrating the presence of their eggs in fecal samples has required the microscopic examination after flotation or sedimentation of certain amount of samples. In many cases, differentiation of eggs from closely related parasites is so difficult. However, molecular diagnostic tools have recently enabled precise diagnosis of even phylogenetically related species (22).

In accordance with the above-mentioned allegation, the liver flukes are amongst the same endoparasites (2) were detailed, “*F. hepatica* has both hermaphroditic and hibrid form. The adult forms of fluke can produce hybrid eggs having a more vast diversity compared to the adult forms. Nevertheless, because the variation with genetic characterization has been studied only in adults, eggs excreted in the feces represent the following stage and generation responsible for infecting hosts. Therefore, the study of genetic variability on eggs would allow predicting the evolution of it within a population of hosts”.

As in many parts of the world, several field studies in Iran have been conducted on animals and human fascioliasis throughout the country. Most of those studies have been based on either the identification of the adult forms of *Fasciola* species from slaughterhouses based on molecular diagnosis (4, 5, 12, 14–17) or the determination of the seroprevalence of fascioliasis in human communities (29, 30–33). Nevertheless, no field study has discriminated *Fasciola* species eggs from living ruminants in Iran. However, in a few studies based on coprological examination, 32.1% of cattle and 32% of sheep from animals of northern Iran (34), 32% of cattle from Guilan Province (35) and 32.5, 12.1 and 3.1% of cattle, and also 9.53, 7.8 and 2.5% of sheep from Guilan, Mazandaran and Golestan provinces, respectively (34) have been reported to be infected with *Fasciola* eggs. The *Fasciola* eggs were solely identified on genus level, whereas in the present study molecular diagnostic technique such as PCR-RFLP has been applied for the species identification, which is a reliable, rapid, specific and more accurate diagnostic tool. Indeed, extracting DNA from *Fasciola* eggs and discriminating *Fasciola* species in the present study for the first time was a novelty from this point of view.

Findings of the current study are in accordance with the studies (1, 36) from Pakistan, where PCR with DSJF/DSJ3 primers were used to identify *F. hepatica* eggs from fecal samples of naturally infected various domestic ruminants and accordingly, PCR was found to be more powerful diagnostic tool to detect *Fasciola* infection.

In a similar work, in order to follow up anthelmintic resistance (AR), *F. hepatica* infection in feces of naturally infected sheep flocks from Spain could be detected by developing a PCR technique based on amplification of a 292-bp fragment of ITS2 (11). Likewise, in this country, through amplifying and sequencing the fragments of cytochrome C oxidase subunit 1 and NADH subunit 1 genes to detect AR in *F. hepatica* from sheep three varieties of fluke were characterized in terms of resistance degrees (2). Recently, the advantage of PCR in feces was compared to fecal egg count (FEC) and Sandwich-ELISA methods for the early diagnosis of the *F. hepatica* in sheep infected experimentally as well as naturally (21).

In the current study, the identity of all recovered *Fasciola* eggs from fecal samples was only confirmed by the PCR-RFLP analysis as *F. hepatica* species due to the specific 529-bp bands. The results from PCR-RFLP analysis of current study were similar to findings from an investigation carried out in Spain (27), demonstrating the ability of mentioned technique to identify *Fasciola* species in fecal specimens from certain geographical regions. Moreover, in northwestern Iran (Tabriz), with application of PCR-RFLP of ITS1 region on rDNA, the adult flukes of *F. gigantica* and *F. hepatica* removed from the involved livers of slaughtered sheep and cattle could be differentiated (14). Besides, in the same area of Iran, (18) PCR-RFLP of internal transcribed spacer (ITS1, 5.8S rDNA, ITS2) was reported that the *Fasciola* flukes removed from the affected livers of sheep and cattle belonged to *F. hepatica* only. Due to this discrepancy and the similarity in findings, further investigations are suggested. Furthermore, because of no anthelmintic resistance studies in Iran, this molecular method could be applied to survey the efficiency of anthelmintics after treatment.

## Conclusion

The PCR method is proper for most epidemiological surveys. The findings of this study based on PCR-RFLP indicated that *F. hepatica* was dominant species in animals of studied areas and no evidence of *F. gigantica* was observed. Therefore, further field studies to verify these results are suggested.
